# When a Little Bit More Makes the Difference: Expression Levels of GKRP Determines the Subcellular Localization of GK in Tanycytes

**DOI:** 10.3389/fnins.2019.00275

**Published:** 2019-03-29

**Authors:** Magdiel Salgado, Patricio Ordenes, Marcos Villagra, Elena Uribe, María de los Angeles García-Robles, Estefanía Tarifeño-Saldivia

**Affiliations:** ^1^Department of Cellular Biology, Faculty of Biological Sciences, University of Concepción, Concepción, Chile; ^2^Department of Biochemistry and Molecular Biology, Faculty of Biological Sciences, University of Concepción, Concepción, Chile

**Keywords:** tanycytes, metabolic, glucokinase, GK regulatory protein, glucosensing

## Abstract

Glucose homeostasis is performed by specialized cells types that detect and respond to changes in systemic glucose concentration. Hepatocytes, β-cells and hypothalamic tanycytes are part of the glucosensor cell types, which express several proteins involved in the glucose sensing mechanism such as GLUT2, Glucokinase (GK) and Glucokinase regulatory protein (GKRP). GK catalyzes the phosphorylation of glucose to glucose-6-phosphate (G-6P), and its activity and subcellular localization are regulated by GKRP. In liver, when glucose concentration is low, GKRP binds to GK holding it in the nucleus, while the rise in glucose concentration induces a rapid export of GK from the nucleus to the cytoplasm. In contrast, hypothalamic tanycytes display inverse compartmentalization dynamic in response to glucose: a rise in the glucose concentration drives nuclear compartmentalization of GK. The underlying mechanism responsible for differential GK subcellular localization in tanycytes has not been described yet. However, it has been suggested that relative expression between GK and GKRP might play a role. To study the effects of GKRP expression levels in the subcellular localization of GK, we used insulinoma 832/13 cells and hypothalamic tanycytes to overexpress the tanycytic sequences of *Gckr*. By immunocytochemistry and Western blot analysis, we observed that overexpression of GKRP, independently of the cellular context, turns GK localization to a liver-like fashion, as GK is mainly localized in the nucleus in response to low glucose. Evaluating the expression levels of GKRP in relation to GK through RT-qPCR, suggest that excess of GKRP might influence the pattern of GK subcellular localization. In this sense, we propose that the low expression of GKRP (in relation to GK) observed in tanycytes is responsible, at least in part, for the compartmentalization pattern observed in this cell type. Since GKRP behaves as a GK inhibitor, the regulation of GKRP expression levels or activity in tanycytes could be used as a therapeutic target to regulate the glucosensing activity of these cells and consequently to regulate feeding behavior.

## Introduction

The glucose homeostasis depends on specified cell types capable of detecting and respond to changes in systemic glucose concentration (Marty et al., 2007; Thorens, 2015). Such cell types so-called glucose’s sensors, are provided with a molecular machinery allowing them to efficiently incorporate and metabolize glucose (Yang et al., 1999; Routh, 2002; Prentki et al., 2013). Several proteins have been involved in the glucose sensing mechanism, being the most studied GK (Matschinsky, 1990; Schuit et al., 2001; Printz and Granner, 2005), glucose transporter 2 (GLUT2) (Schuit et al., 2001; Thorens, 2015) and the GKRP (Printz and Granner, 2005). GK or Hexokinase IV is an isoenzyme belonging to the hexokinases family (Wilson, 1995), enzymes catalyzing the phosphorylation of glucose to G-6P using ATP as cofactor (Cárdenas et al., 1998; Iynedjian, 2009; Lenzen, 2014). GK, encoded by the *Gck* gene, is a protein of 52 kDa with a high K_m_ to its subtract (K_m_ 5–12 mM), it is not inhibited by product (Matschinsky, 2002), and its activity increases concomitantly with rising of blood glucose displaying great capacity to phosphorylate glucose in a wide range of physiological conditions. GK was first found and characterized in liver (Sharma et al., 1963), but later it has been detected in several glucosensor tissue/cell types such as in pancreas (Iynedjian et al., 1986), pituitary (Hughes et al., 1991; Magnuson, 1992), hypothalamus and hypothalamic tanycytes (Navarro et al., 1996; Roncero et al., 2000; Alvarez et al., 2002; Millán et al., 2010; Orellana et al., 2012; Salgado et al., 2014). In liver, GK activity is regulated by GKRP, a protein of 69 kDa that acts as a regulator of GK activity and localization (Van Schaftingen et al., 1994; Baltrusch and Tiedge, 2006) and encoded by *Gckr* gene. On one hand, GKRP binds GK acting as a competitive inhibitor by decreasing its affinity for glucose (Van Schaftingen, 1989). GKRP, in turn, is activated by fructose-6-phosphate stabilizing the complex, while fructose-1-phosphate release inhibition of GK and destabilize GKRP-GK interaction (Pautsch et al., 2013). On the other hand, GKRP seems to play an important role in nuclear translocation and nuclear sequestration of GK in response to glucose variations (Brown et al., 1997; Chu et al., 2004). GKRP has been detected in liver, hypothalamus, and tanycytes (Brown et al., 1997; Salgado et al., 2014). In liver, when glucose concentration is low, GKRP binds to GK holding it in the nucleus, while rise in glucose concentration induces a rapid export of GK from the nucleus to the cytoplasm in which GKRP is also exported (Toyoda et al., 1994, 1995; Brown et al., 1997; Mukhtar et al., 1999). Nuclear translocation of GK has been suggested as a mechanism to allow the release of glucose in hypoglycemia, from glycogenolysis, by the liver avoiding glucose/G-6P futile cycle (Brown et al., 1997). In contrast, with high glucose concentrations, translocation of GK to the cytoplasm is needed for the enzyme to exert his catalytic function.

Several groups have suggested that subcellular localization of GK and GKRP is mutually dependent (Bosco et al., 2000). For example, pancreatic beta cells do not express GKRP and do not display nuclear localization of GK (Noma et al., 1996; Toyoda et al., 1999). Furthermore, overexpression of GK and GKRP by themselves in HeLa and 293T cells (no glucose sensor systems) result on cytoplasmic localization of GK and cytoplasmic and nuclear localization for GKRP, while overexpression of both proteins together determines a nuclear distribution of GK and GKRP (Shiota et al., 1999; Bosco et al., 2000). Additionally, overexpression of GKRP in insulinoma cells, which contain endogenous GK but not GKRP, induce nuclear localization of GK (Bosco et al., 2000). Likewise, GKRP-KO mice display cytoplasmic distribution of GK independent of glucose or F6P concentrations (Farrelly et al., 1999) in liver. Thus, GKRP causes GK to change subcellular distribution as GK have not the capacity to enter to the nucleus alone.

Inverse nuclear compartmentalization dynamic in response to glucose concentration has been shown for our group (Salgado et al., 2014). In hypothalamus and primary cultures of hypothalamic tanycytes, a rise from 0.5 to 15 mM glucose drives nuclear compartmentalization of GK, opposite to what has been shown for the liver so far. Hypothalamic tanycytes are glial cells, similar to beta pancreatic cells, expressing the pancreatic GK isoform, GLUT2 and an isoform of GKRP similar to liver (Millán et al., 2010; Salgado et al., 2014). In this cell type, expression of GKRP has been detected at shallow levels, however, subcellular localization of GKRP was documented both on nucleus and cytoplasm (Salgado et al., 2014), which has been described in liver (Mukhtar et al., 1999). Based on this observation, the compartmentalization dynamics of GK in response to glucose might be determined by the cellular context and metabolic conditions together with than presence of GKRP. In order to understand better the GK and GKRP compartmentalization dynamics observed in tanycytes, we studied the effects of overexpress of *Gckr* cloned from tanycytes in a cellular model, similar to tanycytes, that is responsive to glucose and express GK; the insulinoma INS-1 (832/13) cell line. First, we characterize the expression of glucosensing genes by different molecular approaches and concluded that insulinoma cells are a GKRP-free cell system. Then, we evaluated how GKRP overexpression affects subcellular localization of GK in response to dynamical variations of glucose, concluding that overexpression of GKRP induces nuclear localization of pancreatic GK in response to low glucose concentration, following a dynamic similar to hepatocytes. Based on tanycytes display an inverse GK compartmentalization dynamics, we evaluated the effects of tanycytic *Gckr* overexpression in response to glucose in primary tanycyte cultures. We observed that overexpression of GKRP, turns GK localization to a liver-like fashion, as GK is mainly localized in the nucleus in response to low glucose in tanycytes. Evaluating the expression levels of GKRP in relation to GK, by calculating the ratio *Gckr/Gk* mRNA expression, we observed that excess of GKRP might influence the pattern of GK subcellular localization in response to a metabolic condition. Since GKRP behaves as a GK inhibitor, the regulation of GKRP expression levels or activity in tanycytes could be used as a therapeutic target to regulate the glucosensing activity of these cells.

## Materials and Methods

### Ethics Statement

All animals were handled in accordance with the Animal Welfare Assurance, and all animal work was approved by the appropriate Ethics and Animal Care and Use Committee of the Universidad de Concepcion, Chile. Adult male Sprague-Dawley rats weighing 200–300 g were used for the experiments. Animals were housed in a room to 21 ± 2°C and a 12 h light/12 h-dark cycle was turned on every day at 7:00 a.m. Animals had free access to a standard rodent diet (Lab Diet, 5P00 Prolab RMH 3000, Purina Mills, St. Louis, MO, United States) and water.

### Primary Culture of Tanycytes

Hypothalamic tanycyte cultures from 1-day postnatal brains (12–16 rats) were isolated following the method described previously (García et al., 2003; Cortés-Campos et al., 2011; Orellana et al., 2012; Salgado et al., 2014). Briefly, the hypothalamic region was removed from the brain and further dissected to obtain the tissue containing the ependymal layer. After the tissue was subjected to enzymatic disaggregation for 30 min at 37°C in 0.25% trypsin (Invitrogen, Rockville, MD, United States) and EDTA 0.20% (Sigma-Aldrich, St. Louis, MO, United States). Subsequently the tissue was transferred to culture plates containing MEM medium (Invitrogen, Carlsbad, CA, United States) with 10% (v/v) fetal bovine serum (FBS) (Thermo Fisher Scientific Inc., Waltham, MA, United States) and 2 mg/mL DNase I (Sigma-Aldrich, St. Louis, MO, United States). Cells were seeded in culture dishes treated with 0.2 mg/mL poly-L-lysine (Sigma-Aldrich). After 4 h, the culture medium was changed to MEM (5 mM glucose) supplemented with 10% FBS, 2 mM L-glutamine, 100 U/mL penicillin, 100 mg/mL streptomycin, and 2.5 mg/mL fungizone (Thermo Fisher Scientific, Inc.). Cells were cultured in the same dish for 2 weeks, and the medium was changed every 2 days. Dishes were expanded for adenoviral transduction. To determinate the effect of glucose on the intracellular localization of GK and GKRP, cells were grown in glucose-free DMEM supplemented with 2% FBS and 0.5 mM glucose for 6 h and were subsequently supplemented with 15 mM glucose for 30 and 60 min before immunocytochemistry analyses.

### INS-1 832/13 Culture

The rat INS-1-derived cell line, 832/13 (Hohmeier et al., 2000) (kindly provided by Dr. Thomas Becker, from Duke University) was maintained at 37°C and 5% CO_2_ on Petri dishes (Falcon) with 10 mL of RPMI-1640 culture medium (Gibco BRL) containing 10% v/v serum fetal bovine (SBF) (Gibco BRL), 10 mM Hepes, 2 mM L-glutamine, 1 mM sodium pyruvate, 0.05 mM β-mercaptoethanol, 1000 IU/mL penicillin, 100 μg/mL streptomycin and fungizone 2.5 μg/mL (Gibco BRL). The dishes with the highest density of confluent cells were expanded (1:5) and used for adenoviral transduction, RT-PCR, Western blot, immunocytochemistry and GK activity determination. To determinate the effect of glucose on the intracellular localization of GK and GKRP, cells were grown in glucose-free DMEM supplemented with 2% FBS and 0.5 mM glucose for 6 h and were subsequently supplemented with 50 mM glucose for 30 min before immunocytochemical analyses.

### Reverse Transcription-Polymerase Chain Reaction (RT-PCR)

Total RNA was isolated from liver, hypothalamus, pancreas, tanycyte primary cultures and 832/13 culture using Trizol (Invitrogen). The RT-PCR was performed according to the manufacturer’s protocol using 2 μg RNA (Fermentas International INC.). Parallel reactions were performed in the absence of reverse transcriptase to control for the presence of contaminant genomic DNA. The PCR reaction was performed using 1 μL cDNA and the following sets of primers: GK, sense (NM_012565) 5′ ATG GCT ATG GAT ACT ACA AGG TGT G 3′ and antisense 5′ TGC ATT CAG AGA TGT AGT CAA AGA G 3′ (expected product of 388 bp); GKRP (X68497.1), sense 5′ AGA CAG AAG ATA GCG CCC TAC ACG 3′ and antisense 5′ CTT TGA GAG GAC ACA ACA CCC TGG 3′ (expected product 418 bp); GLUT2 (NM_012879.2), sense 5′ GGC TAA TTT CAG GAC TGG TT 3′ and antisense 5′ TTT CTT TGC CCT GAC TTC CT 3′ and β-actin (NM_031144.3), sense 5′ GCT GCT CGT CGA CAA CGG CTC and antisense 5′ CAA ACA TGA TCT GGG TCA TCT TCT C 3′ (expected product 353 bp). Each reaction mixture was incubated at 95°C for 5 min followed by 35 cycles of 30 s at 95°C, 30 s at 55°C, and 30 s at 72°C and a final extension of 7 min at 72°C. PCR products were separated by 1.2% agarose gel electrophoresis and visualized by staining with ethidium bromide.

### Quantitative PCR

RT-qPCR analysis was used to measure the expression of *cyclophilin*, *Gckr* and *Gck* from liver, pancreas, basal hypothalamus, primary tanycyte cultures, and insulinoma cells. The following sets of primers were used: cyclophilin, sense 5′-ATA ATG GCA CTG GTG GCA AGT C-3′ and antisense 5′-ATT CCT GGA CCC AAA ACG CTC C-3′; Gckr, sense 5′-TGG TGA ATG GGA GTT GTC AGG GTA-3′ and antisense 5′-TTC CAG CCA CTT GCA ACA TGG T-3′; Gck, sense 5′-TGT GAG GCA CGA AGA CCT AGA CAA-3′ and antisense 5′-ACC AGC TCC ACA TTC TGC ATT TCC-3′. First, total RNA from rat tissues was isolated by using Trizol^®^ reagent. The reverse transcription reaction was performed according to the manufacturer’s protocol of M-MULV reverse transcriptase (Fermentas International INC.). PCR reactions were carried out in an Mx3000P QPCR System (Agilent Technologies, Santa Clara, CA, United States). RT-qPCR was performed using the qPCR Master Mix kit for Brilliant II SYBR Green (Agilent Technologies, Inc.) in a final volume of 12.5 μL consisting of 1x SYBR green Master Mix, 0.5 μM of each primer and 1 μL of cDNA sample. All reactions were performed with an initial denaturation of 10 min at 95°C, followed by 40 cycles of 15 s at 95°C, annealing for 15 s at 55°C, and extension for 15 s at 72°C. The relative expression of *Gckr* or *Gck* to *cyclophilin* mRNA was calculated based on the PCR efficiency.

### Immunocytochemistry

Cultured cells were grown on poly-L-lysine-coated (Sigma- Aldrich, St. Louis, MO, United States) glass cover slides in 24-well plates, fixed with 4% paraformaldehyde in PBS for 30 min, washed with Tris–HCl buffer (pH 7.8) containing 8.4 mM sodium phosphate, 3.5 mM potassium phosphate and 120 mM NaCl, and incubated in the same buffer containing 1% bovine serum albumin (BSA) and 0.2% Triton X- 100 for 10 min. Samples were then incubated with the following primary antibodies overnight at room temperature: rabbit anti-GK (1:100, sc 7908; Santa Cruz Biotechnology), rabbit anti-GKRP (1:100, sc-11416; Santa Cruz Biotechnology). Subsequently, cells were incubated with Cy2- or Cy3 -labeled secondary antibodies (Jackson ImmunoResearch Laboratories), counter stained with the DNA stain, TOPRO-3 (1:1000, Invitrogen), and analyzed using confocal laser microscopy Carl Zeiss, LSM700).

### Image Analysis

Digital images were analyzed using ImageJ image analysis software (National Institutes of Health, Bethesda, MD, United States)^1^. For nuclear quantification of GK and GKRP, color channels were separated, and the regions of interest (ROIs) were selected using the nuclear marker channel, by manually outlining the ROI using drawing tools included in the software. Data from 100 nuclei were combined, and the mean and standard deviation for each condition were obtained using GraphPad Prism 5.0 software (GraphPad Software Inc., San Diego, CA, United States).

### Preparations of Adenovirus Particles Expressing GKRP (AdGKRP-RFP) and shGKRP (AdshGKRP)

Serotype 5 ΔE1, E3 based replication-deficient adenoviruses were generated as previously described (Elizondo-Vega et al., 2016; Uranga et al., 2017). To produce adenoviruses capable to overexpress GKRP, we produced GKRP-RFP cassette by incorporating both BamHI and KpnI at the 5′-and-3′ end of tanycyte GKRP cDNA to clone it into the adenoviral shuttle vector (Supplementary Figure 1). To produce AdshGKRP, oligonucleotides targeting rat GKRP were designed and selected using the Genebank accession number KJ026952.1, sense shRNA-GKRP 5′-CGC GCC GCC AAA GCA GAT GCA GAG AAA T-3′ and antisense shRNA-GKRP 5′-TTA AAA AAA CAA AGC AGA TGC AGA GAA A-3′. Each shuttling plasmid was then co-transfected with the Ad genomic plasmid, pBHGloxΔE1,3Cre (Admax system, Microbix Biosystems, Mississauga, ON, Canada) into HEK293A cells. Virus particles were released by heat shock, and cell debris was removed by centrifugation for 5 min at 5000 ×*g*. The particles were recovered from the supernatant by filtration through a 0.45-μm filter. The resulting adenoviral particles were tittered by RFP expression using the Adeno-XTM Rapid Titer Kit Protocol (Clontech, Mountain View, CA, United States) and stored at -80°C.

### Overexpression of GKRP and GK Location Dynamics

To determine GK translocation in response to glucose, 832/13 cells were grown on poly-L-lysine-coated glass cover slides in 24-well plates and then were transduced with 5 × 10^7^ ifu/mL of AdGKRP-RFP for 72 h. As transduction control, we used an RFP-expressing adenovirus (Ad-Control). After that, cells were incubated with 3 mM glucose for 3 h and then with 30 mM glucose for 35 min, or vice versa. We choose 30 mM glucose for simulating hyperglycemic condition in culture, and not 50 mM glucose, to achieve glucose concentrations closer to physiological conditions and avoid hypertonicity-related effects. All glucose stimuli were dissolved in HBSS buffer (114 mM NaCl, 4.7 mM KCl, 1.2 mM KH_2_PO_4_, 1.16 mM MgSO_4_, 20 mM HEPES, 2.5 mM CaCl_2_, 25.5 mM NaHCO_3_, pH 7.2). Saccharose was used as an osmolarity control. Cells were washed with PBS and immediately fixed with PFA every 5 min until 35 min. The glucose changes were used as initial point (t_0_) in each condition. After cells permeabilization, cells were processed for immunocytochemistry by using anti-GK antibody.

### Immunoblotting

Total protein extracts were obtained from rat liver, pancreas, periventricular hypothalamus, insulinoma cells, and tanycyte cultures and by homogenizing the tissue or cells in buffer A (0.3 mM sucrose, 3 mM DTT, 1 mM EDTA, 100 mg/mL PMSF, 2 mg/mL pepstatin A, 2 mg/mL leucopeptin, and 2 mg/mL aprotinin). The periventricular hypothalamus was obtained from fresh ice-cold brains by making two transverse cuts, one at the optic chiasm and another just before the mammillary bodies, dissecting the area closest to the diencephalic third ventricle. Subsequently, the samples were sonicated on ice at 300 W (Sonics & Material INC., VCF1, Newtown, CT, United States) 3 times for 10 s. After centrifugation at 8000 *g* for 10 min, supernatants proteins were resolved by SDS-PAGE (50 or 100 μg/lane) in a 10% (w/v) polyacrylamide gel, transferred to PVDF membranes (0.45 μm pore, Amersham Pharmacia Biotech., Piscataway, NJ, United States), and probed with rabbit anti-GK, anti-GKRP, anti-lamin B1 and anti-β-actin antibodies. After extensive washing, the PVDF membranes were incubated for 2 h at 4°C with peroxidase-labeled anti-rabbit IgG (1:5000; Jackson Immuno Research). The reaction was developed using the enhanced chemiluminescence (ECL) Western blotting analysis system (Amersham Biosciences). Negative controls consisted of incubating the membrane with a pre-absorbed antibody (anti-GK 1:1000 with 1 mg/mL inductor peptide incubated at 4°C overnight), or the absence of anti-GKRP.

### Nuclear Extract Preparation

To obtain nuclear extracts from insulinoma cells, we used NE-PER Nuclear and Cytoplasmic Extraction Reagents (Thermo Scientific, Waltham, MA, United States) following the manufacturer’s instructions. All procedures following the cell disruption were performed on ice or at 4°C. The purity of the nuclear extracts was confirmed by western blot analysis using anti-lamin B1 antibody (ab16048, Abcam, Cambridge, England, United Kingdom), a nuclear marker.

### Enzyme Assays

To evaluate the effect of GKRP overexpression over GK activity, 832/13 cells were transduced with 5 × 10^7^ ifu/mL AdGKRP or Ad-Control and incubated for 72 h. After that, cells were lysed by ultrasound and promptly HK activity was determined as previously described with slight modifications (Salgado et al., 2014). Briefly, a G-6P dehydrogenase-coupled reaction was used, and the activity was followed by measuring the increase in absorbance at 340 nm after 5 min incubation at 37°C. The reaction mixture consisted of 200mM Tris-HCl buffer (pH 7.5), 2mM MgCl_2_, 1mM DTT, 1 mM ATP, 0.5 mM NADP^+^, 1–30 mM glucose, and 1 U/mL of G-6P dehydrogenase (Sigma-Aldrich). For specific activity determination, we used 0.5 mg/mL of total protein in the reaction mixture and the Prism software was used for data analysis (GraphPad, Inc.). To determine velocity reaction for each absorbance, we made a calibration curve with G-6P as substrate.

## Results

### Rat Insulinoma Cell Line Does Not Express GKRP and Does Not Compartmentalize Glucokinase in Response to Glucose

**FIGURE 1:**
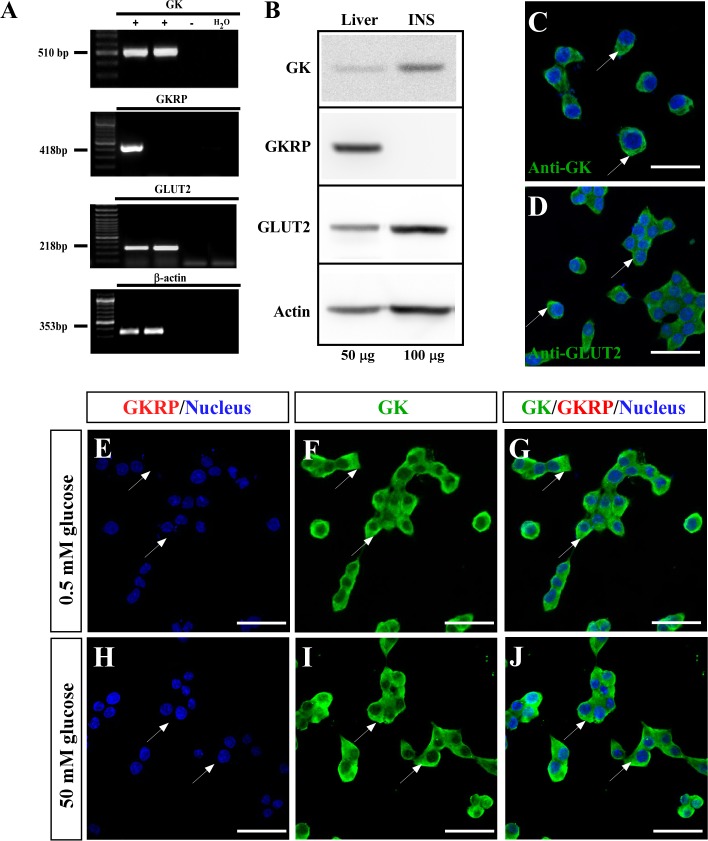
Expression and subcellular localization of glucose sensor machinery in insulinoma cells. **(A)** RT-PCR for GK (510 bp; first panel), GKRP (418 bp; second panel), GLUT2 (218 bp third panel), and β-actin (353 bp; fourth panel). PCR products were amplified from liver (lane 1), 832/13 culture (lane 2), negative control (RT -) of liver (line 3) and water (lane 4). **(B)** Western Blot from total protein extracted from liver (lane 1, liver, 50 μg of protein loaded) and 832/13 culture (lane 2, INS, 100 μg of protein loaded). Immunodetection was performed for GK (first panel, 52 kDa), GKRP (second panel, 69 kDa), GLUT2 (third panel, 60 Kda), and β-actin (fourth panel, 43 kDa). **(C)** Immunocytochemistry for GK. **(D)** Immunocytochemistry for GLUT2. **(E–G)** Immunocytochemistry for GK and GKRP in insulinoma cultured at 0.5 mM glucose and **(H,I)** 50 mM glucose. **(E,H)** Immunostaining for GKRP and nucleus, **(F,I)** GK, and **(G,J)** Merge. Nuclear staining performed by Hoechst. Scale bar: 50 μm. White arrows are indicating the cytoplasm of insulinoma cells.

As previously shown by several groups (Hohmeier et al., 2000; Millán et al., 2010), the INS-1-derived cell line (832/13), called herein insulinoma, expressed key components of the glucose sensing machinery such as GLUT2 and GK, as well as they are responsive to glucose by insulin secretion at physiological range. We aimed to characterize gene expression on this cell line, to evaluate compartmentalization of GK in response to glucose. As expected, we detected high expression of GK and GLUT2 both at the mRNA and protein level (Figure 1A–D). As insulinoma cells have a pancreatic origin (beta cells), we investigated whether express GKRP. We performed RT-PCR (Figure 1A), western blot (Figure 1B), and immunocytochemistry (Figure 1C,D), with no detection of GKRP even loading twice amount of proteins for insulinoma cells in the western blot assays (Figure 1B). Thus, as GKRP is not expressed on these cells, we did not expect to observe nuclear localization of glucokinase in response to glucose. To evaluate this, we performed immunocytochemistry for GK (including immunostaining of GKRP) of insulinoma cell cultured with 0.5 and 50 mM glucose for 30 min (Figure 1E–J). As shown in Figure 1F,I, we did not observe nuclear staining for GK with neither of glucose concentration. This result indicates that, as observed on pancreatic beta cells, insulinoma cells do not display nuclear localization of GK in response to glucose.

### Overexpression of GKRP in INS-1 Cell Lines Determines a Liver-Like Compartmentalization of GK in Response to Glucose

**FIGURE 2:**
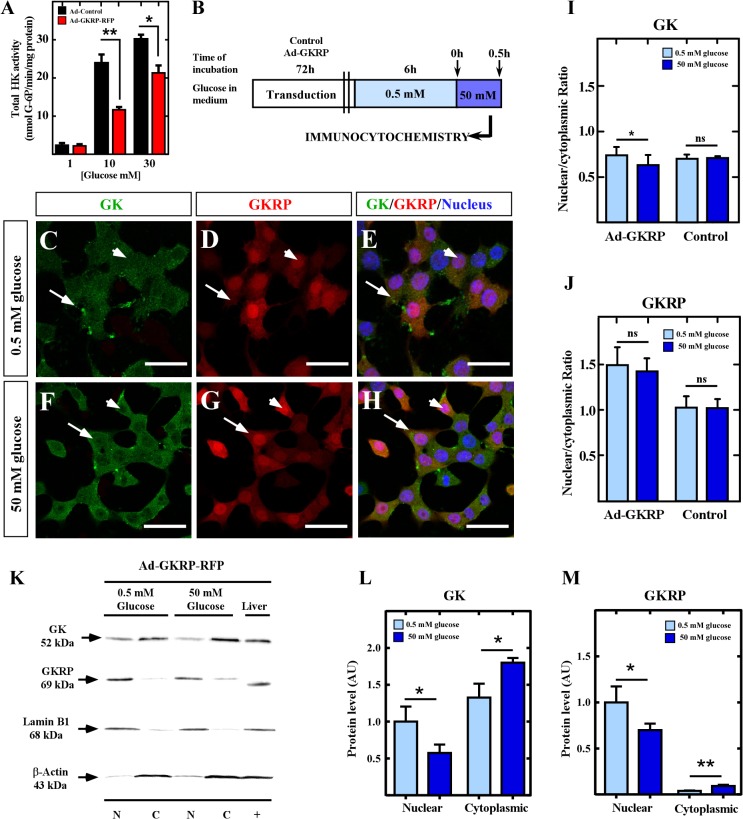
Subcellular localization of GK and GKRP in insulinoma cells overexpressing GKRP. **(A)** Total hexokinase activity measured at 1, 10, and 30 mM glucose on Ad-GKRP (red bars) and Ad-Control (black bars). **(B)** Schema of glucose treatments for transduced cells to evaluate compartmentalization dynamics. **(C,D)** Immunocytochemistry for GK **(C,F)** and GKRP **(D,G)** of cells cultured at 0.5 mM **(C–E)** and 50 mM **(F–H)** glucose. **(I,J)** Quantification of nuclear and cytoplasmic fluorescence for GK **(I)** and GKRP **(J)** immunocytochemistry. **(K)** Immunodetection by western blot of GK (52 kDa, first panel) and GKRP (69 kDa, second panel) in nuclear and cytoplasmic protein extracts of transduced insulinoma cultured at 0.5 and 50 mM glucose. Lamin B1 (68 kDa, third panel) and *β*-actin (43 kDa, fourth panel) were used to confirm the purity of extracts and correct nuclear and cytoplasmic quantification. **(L,M)** Densitometry analysis of GK **(L)** and GKRP **(M)** at both glucose concentrations. Statistical *T*-test, ^∗^*p*-value < 0.05, ^∗∗^*p*-value < 0.01. White arrows are indicating the cytoplasm of insulinoma cells, while arrowheads indicate the nucleus.

In liver, expression of GKRP induces sequestration of GK to the nucleus in response to low glucose concentrations. Furthermore, we have shown that nuclear localization, triggered by changes in glucose concentrations, will depend on the cell type as it can be detected either on hypoglycemia (in liver) or hyperglycemia (in tanycytes) at the same time in the same animal (Salgado et al., 2014). As we have confirmed that insulinoma cells are a cellular system free of GKRP, we wonder which patter of compartmentalization of GK can be observed when overexpression of GKRP is induced on this cell line incubated at low and high glucose. To achieve overexpression of GKRP in insulinoma cells, we constructed the adenovirus (Ad-GKRP) that overexpressed the fusion protein GKRP-RFP (*Gckr* sequence cloned from tanycytes; Salgado et al., 2014) under the control of human ubiquitin promoter (Supplementary Figure 1). We infected insulinoma cells with Ad-GKRP or Ad-control and let cell recovered for 72 h. First, we confirmed that exogenous GKRP was capable of inhibiting GK activity (Figure 2A) by measuring the amount of G-6P produced under different glucose concentration. Proteins extracted from transduced insulinoma cells were incubated with 1, 10, or 30 mM glucose for 5 min, and total hexokinase activity was assessed by measuring the increase in absorbance at 340 nm as an indicator of G-6P produced (see methods). At 1 mM glucose, when hexokinase I-III (HKs) have a high contribution at the phosphorylation activity, we did not observe any change in the hexokinase activity between Ad-control and Ad-GKRP transduced cells, as expected since it has been shown that GKRP does not inhibits HKs (Van Schaftingen et al., 1992). When we used 10mM glucose, where HKs are saturated and the contribution of GK to the phosphorylating activity is higher, we observed a highly significant decrease on the phosphorylation activity only in Ad-GKRP-transduced cells (Figure 2A). At 30 mM, a glucose concentration close to saturation, we observed a minor inhibition of GK that might be the result of conformational changes in GK induced by high glucose concentrations, these changes generate a minor affinity of GKRP for GK (Choi et al., 2013). These results indicate that overexpressed GKRP, introduced by adenoviral infection in insulinoma cells, is capable of inhibiting GK activity.

Then, we investigated if overexpression of GKRP in insulinoma cells drives nuclear compartmentalization of GK in response to glucose. For that, cells transduced with Ad-GKRP (Figure 2C–H), Ad-Control, and no transduced cells (Supplementary Figure 2) were incubated with 0.5 and 50 mM of glucose for 30 min (Figure 2B), and posteriorly we evaluated subcellular localization of GK and exogenous GKRP by immunocytochemistry. At 0.5 mM glucose, transduced GKRP display predominant nuclear localization (Figure 2D,E, red), while GK display both cytoplasmic and nuclear distribution (Figure 2C,E, green). At 50 mM glucose, we observed translocation from the nucleus to the cytoplasm of both GK and GKRP proteins (Figure 2F,H). Quantification of the nuclear and cytoplasmic fluorescence was performed by ImageJ software, confirming the significant loss of GK nuclear signal and a tendency to exit the nucleus of GKRP at high concentrations of glucose (Figure 2I,J). These results were confirmed by western blots of cytoplasmic and nuclear protein extracts from Ad-GKRP transduced insulinoma cultured at 0.5 or 50 mM glucose, using lamin B1 and β-actin to prove the purity of fractions. As shown in Figure 2K–M, GK and exogenous GKRP increase in cytoplasmic fractions with high glucose concentrations.

**FIGURE 3:**
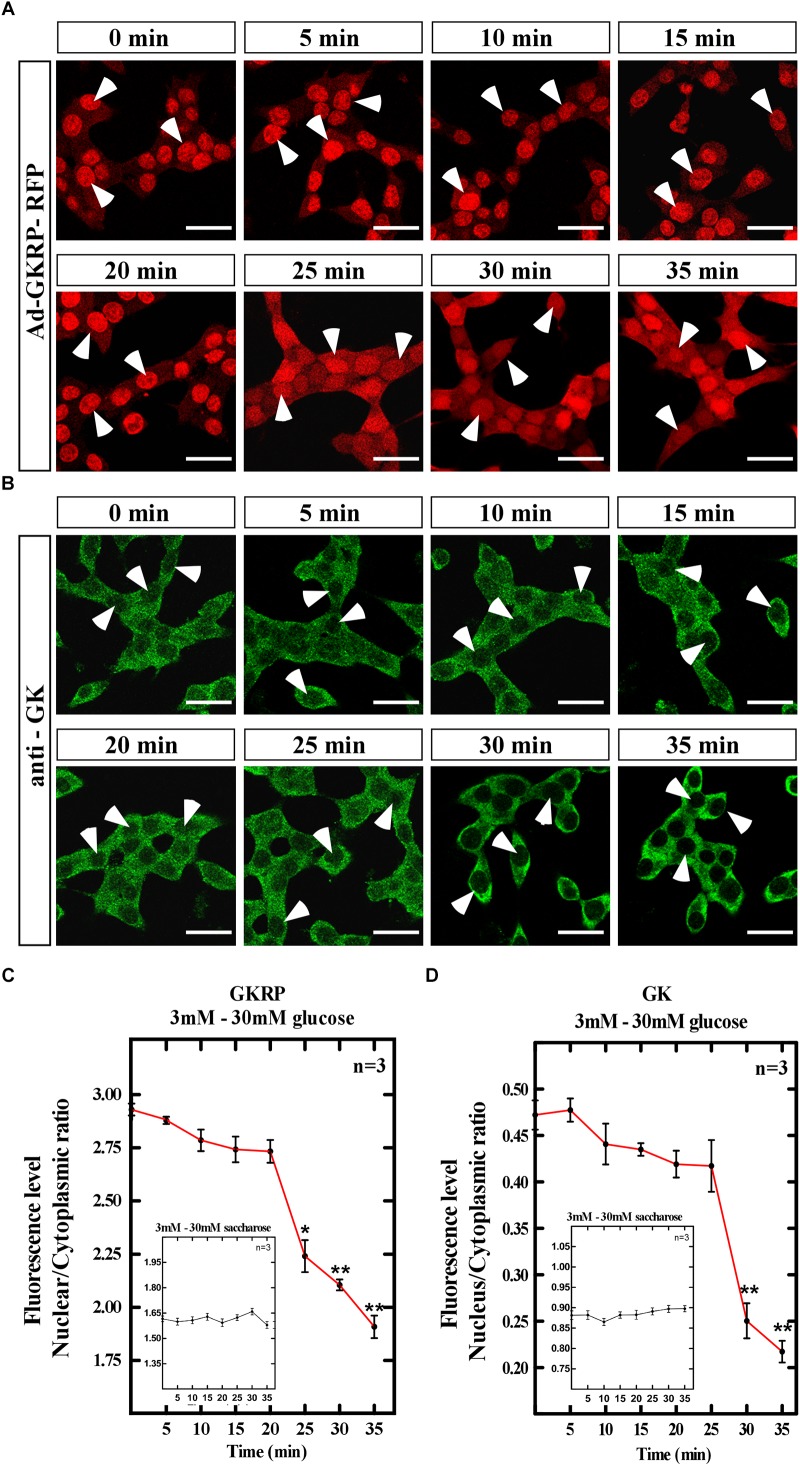
Translocation dynamics for GK and GKRP in insulinoma cells overexpressing GKRP at increasing glucose concentrations. Transducted insulinoma cells were cultured for 3 h at 0.5 mM glucose with a posterior increase to 50 mM glucose for 30 min. **(A)** Dynamic subcellular localization of GKRP through fluorescence detection of RFP. **(B)** Dynamic subcellular localization of GK through immunocytochemistry. Scale Bar 20 μm. **(C,D)** Quantification of nuclear/cytoplasmic ratio measured by fluorescence intensity of both subcellular compartments for GKRP **(C)** and GK **(D)**. Inserted box: Saccharose control treatments. Statistical ANOVA, ^∗^*p*-value < 0.05, ^∗∗^*p*-value < 0.01. Arrowheads show the nucleus of insulinoma cells, indicating nuclear exclusion.

Hepatocytes display a fast response to increasing glucose concentrations leading to GK release from the nucleus to the cytoplasm at 30 min of exposure (Brown et al., 1997). We evaluated the subcellular localization of GK and GKRP through time in Ad-GKRP transduced insulinoma cells when glucose is increased in the medium. Briefly, cells were incubated during 3 h with 3 mM glucose to rise to 30 mM glucose posteriorly. Cells were fixed every 5 min during 35 min to evaluate immunolocalization of GK and RFP fluorescence (GKRP expression) (Figure 3A,B). As control of tonicity and specificity of the assay, we used saccharose at same concentrations and times of incubation. Changes in subcellular localization were quantified through nuclear and cytoplasmic fluorescence (Figure 3C,D, 60 cell /time, *n* = 3). In Figure 3C, we observed that at 3mM glucose (0 min) GKRP is mainly nuclear, but after 5 min of rising glucose, we found a slight increase in the cytoplasmic signal which is stable up to 20 min. At 25 min incubation, we observed a significant increase on the cytoplasmic localization that keeps increasing up to 35 min. It is important to mention that, while we observed an increase in cytoplasmic signal, a strong nuclear signal for GKRP-RFP was visible over all the period. As for GK immunolocalization, at 3 mM glucose (0 min) we observed nuclear and cytoplasmic localization which remain stable up to 25 min after glucose increase (Figure 3D). At 30 min, we found a marked and significant loss of nuclear signal that suggests nuclear exclusion of GK at high glucose concentrations. Cell treated with saccharose in the same conditions did not change GKRP-RFP or GK localization (Figure 3C,D, inserted box). We also evaluate the opposite dynamics of localization when decreasing glucose concentration from 30 mM (incubated 3 h) to 3 mM in the medium of Ad-GKRP transduced insulinoma cells. As expected, the nuclear signal of GKRP-RFP and immunodetection of GK were increased when decreasing glucose concentration displaying a pronounced peak of compartmentalization after 20 min for GKRP and after 25 min for GK (Supplementary Figure 3).

These results together indicate that overexpression of GKRP (sequence from tanycytes) on insulinoma cell lines, with pancreatic beta cells origin, induces a liver-like compartmentalization pattern of GK in response to increasing glucose concentration. Insulinoma cells have a pancreatic genetic background similar to tanycytes cells, however, tanycytes do not display the same translocation pattern. Tanycytes express low levels of GKRP which are sufficient to trigger nuclear compartmentalization of GK, but oppositely to the observed in hepatocytes (Salgado et al., 2014). We wonder if overexpression of GKRP in tanycytes can turn GK compartmentalization dynamics to a liver-like responsive behavior. To investigate this, we overexpressed GKRP in tanycytes using the Ad-GKRP and evaluate the compartmentalization dynamics.

### In Tanycytes, Liver-Like Translocation of GK Depends on the GKRP Expression Levels

Previously, we have shown that tanycytes express the pancreatic GK isoform, low expression of GKRP (99% identity with hepatic isoform) and display nuclear compartmentalization with high concentrations of glucose in an opposite dynamics to the liver (Salgado et al., 2014). It has been demonstrated, in liver as well as non-sensor cell types, that GKRP is essential for nuclear compartmentalization of GK (Farrelly et al., 1999; Shiota et al., 1999; Bosco et al., 2000). Here, we downregulated GKRP in tanycytes using an adenovirus (ad-shGKRP-EGFP) that overexpress an shRNA for GKRP and observed the compartmentalization dynamics of GK in low and high glucose concentrations in tanycyte primary cultures (Supplementary Figure 4). We found that downregulation of GKRP, through shRNA, disrupt the normal nuclear localization of GK at high glucose concentration, confirming that GKRP is also essential for GK compartmentalization in tanycytes.

**FIGURE 4:**
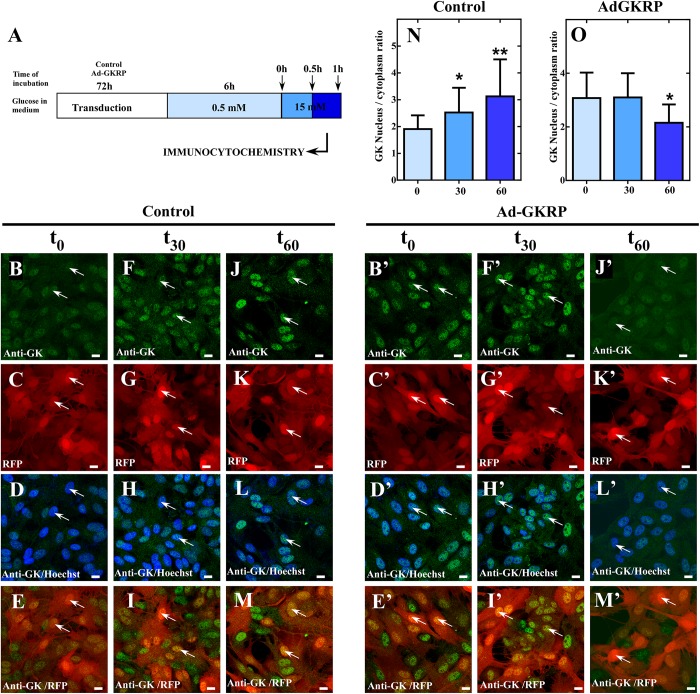
Dynamic localization of GK in culture of tanycytes transduced with Ad-GKRP in response to high glucose. **(A)** Schematic representation of tanycytes transduction and glucose treatment. **(B–M,B’–M’)** Immunocytochemistry for GK in tanycytes transduced with Ad-control **(B–M)** and Ad-GKRP **(B’–M’)** incubated with 15 mM glucose for 0, 30, and 60 min. GK localization (green), Virus transduction (red), and nuclear staining with Hoechst. **(N,O)** Quantification of nuclear/cytoplasmatic ratio of GK fluorescence intensity for Ad-Control **(N)** and Ad-GKRP **(O)**. Statistical *T*-test, ^∗^*p*-value < 0.05, ^∗∗^*p*-value < 0.01. White arrows are indicating the nucleus of tanycytes.

**FIGURE 5:**
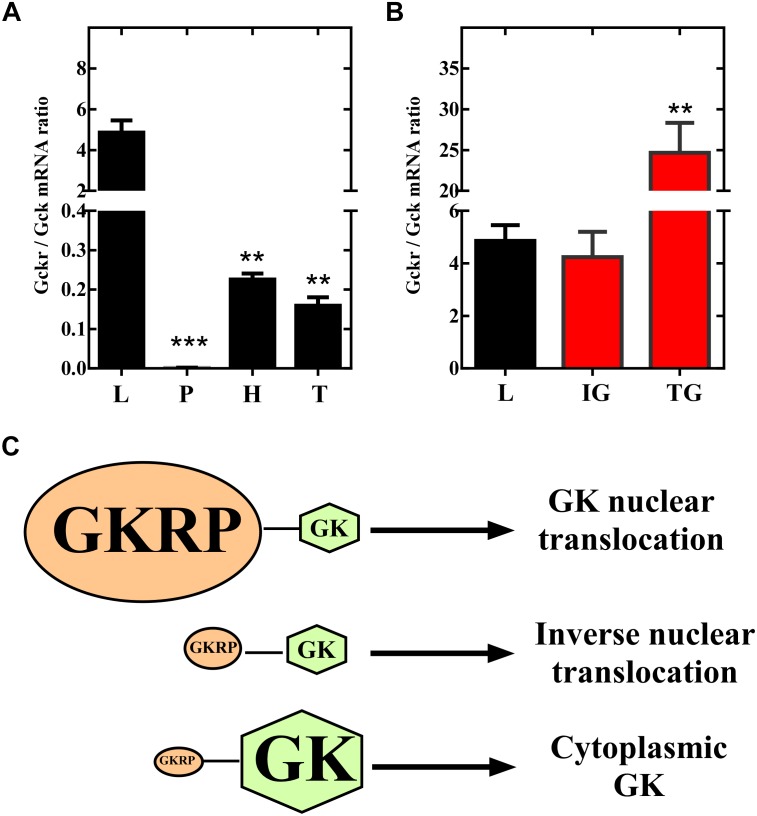
Relative expression of *Gckr* and *Gck* in different tissues. **(A)** Expression levels for *Gckr* and *Gck* were measured by qPCR and expression ratios were calculated for liver (L), pancreas (P), hypothalamus (H), and tanycytes (T). **(B)** Expression ratios for ad-GKRP transduced insulinoma (IG), and ad-GKRP tranduced tanycytes (TG). **(C)** Model to describe translocation dynamics determined by the ratio of expression between Gckr and Gck. When GKRP is in excess, in relation to GK, nuclear translocation is found at low glucose concentrations (as observed in hepatocytes). When GKRP is less expressed, in relation to GK, nuclear translocation is observed at high glucose concentrations (as observed in tanycytes). Statistical *T*-test, ^∗∗^*p*-value < 0.01, ^∗∗∗^*p*-value < 0.001.

Our present results in insulinoma cells, indicate that overexpression of GKRP (sequence from tanycytes) induces a compartmentalization pattern similar to hepatocytes. Thus, we decided to evaluate if overexpression of GKRP in tanycytes can turn the compartmentalization pattern to a liver-like fashion. To do so, primary cultures of hypothalamic tanycytes were infected with the Ad-GKRP and Ad-control and maintained in medium supplemented with 5mM glucose for 72 h. After infection, transduced cells were incubated during 6 h at 0.5 mM glucose to posteriorly increase glucose concentration to 15 mM. After rising glucose, cells were fixed at 0, 30, and 60 min to evaluate subcellular localization of exogenous GKRP through RFP fluorescence and GK by immunolabelling (Figure 4). As previously reported, tanycytes transduced with Ad-control displayed cytoplasmic and nuclear distribution of GK when cultured at 0.5 mM glucose (t_0_) (Figure 4B–E). After 30 min of incubation at 15 mM glucose, tanycytes significantly increase nuclear localization of GK (Figure 4F–I) being higher at 60 min (Figure 4J–M). Surprisingly, tanycytes cultured at 0.5 mM glucose (0 min) that overexpressed GKRP display a mainly nuclear GK localization (Figure 4B’–E’). Indeed, the nuclear distribution is maintained after 30 min of glucose increase (Figure 4F’–I’). At 60 min, we observed a decrease of nuclear localization of GK (Figure 4J’–M’) suggesting that overexpression of GKRP in tanycytes induce a liver-like subcellular translocation of GK in response to increasing glucose concentrations. We estimate the nuclear/cytoplasmic ratio of GK at different incubation periods by quantifying fluorescence intensity of subcellular compartments, through this analysis we confirmed decrease in nuclear signal at high glucose concentration in transduced Ad-GKRP tanycytes (Figure 4N,O). These results suggest that overexpression of GKRP seems to determine the translocation dynamics of GK in response to glucose. A recent study, published by Jin and collaborators (Jin et al., 2015) showed that decreasing GK expression levels to half (using heterozygous Gck w/- mice) lead to cytoplasmic and nuclear localization of GKRP in the liver. If the expression ratio between Gckr and Gck are determinant for the proteins subcellular localization, measuring expression ratios in our experiments might give us some clues to explain our observations. For that, we measured the expression levels of *Gckr* and *Gck* by RT-qPCR in the liver, pancreas, hypothalamus, and tanycytes as well as for transduced insulinoma and, tanycytes cells. Based on the expression levels, we investigated enrichment of *Gckr* over *Gck* as a mean of quantifying the amount of GKRP that is required to induce liver-like compartmentalization (Figure 5). It is important to mention that protein expression of GK and GKRP are not altered in tanycytes cultured for 30 min with high glucose concentration (data not shown). The endogenous *Gckr*/*Gck* ratioshown in Figure 5A indicate that *Gckr* is 5-folds enriched over *Gck* in the liver, conversely, in the hypothalamus and tanycytes the expression of *Gckr* is 5-folds less than *Gk* and in pancreas *Gck* was detected but *Gckr* was absent, for this reason the *Gckr/Gck* ratio tend to zero Surprisingly, when the *Gckr*/*Gck* ratios were evaluated in transduced cultured cells (insulinoma and tanycytes) the relative expression level *Gckr* were similar to liver (Figure 5B). Our observations are summarized in the model included in Figure 5C. These results together might suggest that subcellular localization of GK depend on the expression ratio established between *Gckr/Gck*, as “excess” of GKRP determines nuclear compartmentalization in response to low glucose concentrations in tanycytes. Further functional and molecular characterization must be performed to identify the mechanism driving the particular compartmentalization dynamics in tanycytes.

## Discussion

In the present work, we characterized insulinoma cells as a GKRP free cellular system that displays cytoplasmic distribution of GK independent of glucose concentrations in the medium. These results are in agreement with previous findings that GK has not the capability to enter to the nucleus alone (Noma et al., 1996; Farrelly et al., 1999; Toyoda et al., 1999). Indeed, studies incorporating GKRP and GK exogenously, to cell systems with no glucose sensor abilities, have demonstrated that GKRP is indispensable for GK nuclear translocation (Shiota et al., 1999; Bosco et al., 2000). Here, we used the tanycytic GKRP that share similar kinetic and binding properties that hepatic GKRP (Salgado et al., 2014) for overexpression in insulinoma cells, which induces nuclear localization at low glucose concentrations, as observed in hepatocytes. Our results supported the observation made by Bosco et al. (2000), at low glucose concentrations, in insulinoma cells overexpressing GKRP. However, they did not characterize the dynamics of GK translocation how we performed here, increasing glucose concentrations over the time. Here, we performed a detailed characterization of GK subcellular location in insulinoma cells overexpressing GKRP, switching from low and high glucose concentration, showing that GK is released from the nucleus when glucose concentration increases in the medium. Additionally, we evaluated the compartmentalization dynamics over the time both for GK and GKRP. We observed a very similar pattern of GK compartmentalization to the one observed in the liver (Brown et al., 1997; Chu et al., 2004), as well as, we observed that GKRP exits the nucleus following a similar dynamic than GK (Mukhtar et al., 1999; Shiota et al., 1999). The cytoplasmic localization of GKRP has been controversial, it was thought that GKRP remains in the nucleus to keep GK sequestrated in this compartment when blood glucose is low (Brown et al., 1997; Toyoda et al., 1997; de la Iglesia et al., 1999). However, our results together with others (Mukhtar et al., 1999; Shiota et al., 1999) have confirmed the exit of GKRP from the nucleus. More interesting, the experiments performed in both HeLa and 293T cells (Shiota et al., 1999; Bosco et al., 2000) have demonstrated that GKRP do not require a glucose sensor environment to shuffles from the nucleus to the cytoplasm and that overexpression of GKRP alone also display cytoplasmic and nuclear distribution, suggesting that GKRP do not need GK to be exported to the cytoplasm. In this dynamic, it would be interesting to investigate the mechanism by which GKRP return to the nucleus, as it has been proposed to mediate the GK nuclear entry by a piggy-back mechanism (Shiota et al., 1999) and not NLS has been described for this protein yet.

We have demonstrated, in the same animals, that 30 min of hyperglycemia induces an opposite subcellular localization of GK in liver and hypothalamic tanycytes (Salgado et al., 2014). Then, our results that overexpression of GKRP in tanycytes turns GK translocation dynamics to a liver-like fashion were unexpected for us. Even more, the finding that overexpression of *Gckr* in tanycytes arise higher *Gckr*/*Gck* ratios to the observed usually in liver. Indeed, we obtained that hepatocyte express about 5-folds more *Gckr* than *Gck*, while in tanycytes expression of *Gckr* does not exceed 20% of *Gck* expression (see Figure 5A). Furthermore, our overexpression experiments induced 25-folds increased expression of *Gckr* relative to *Gck* in tanycytes (see Figure 5B). Our results are supported by the work of Jin et al. (2015), using liver-specific GK heterozygous KO mouse displaying slight hyperglycemia, they observed that decreased expression levels of *Gck* (increasing the *Gckr*/*Gck*) lead to increased cytoplasmic localization of GKRP. Unfortunately, this work did not analyze compartmentalization inducing hypoglycemia and hyperglycemia that would allow us to compare with our overexpression analysis in tanycytes. Additionally, our data of *Gckr* downregulation in tanycytes showed that GK nuclear localization induced by high glucose concentration is lost. Taken together, our results strongly suggest that the Gckr/Gck expression ratio might determine, at least in part, the compartmentalization regulated by glucose.

Despite the critical roles of GK and GKRP, the molecular basis for the allosteric regulation mechanism of GK by GKRP remains unclear. Crystallographic structural analysis revealed that GKRP binds to the super-open GK conformation that is more stable in the absence of glucose (Choi et al., 2013). This GK behavior it can be well understood in the liver since GK must be turned off during a fasting state to prevent futile cycling of endogenous glucose to G6P, whereas GK it should be fully active for fast glucose clearance after a meal. Instead, in tanycytes, after 20 min of high glucose concentration, both proteins go to the nuclear compartment. We wonder the purpose of this subcellular dynamic of localization in tanycytes. We and others have postulated that, in response to high glucose, tanycytes release lactate to activate anorexigenic neurons of the AN (Ainscow et al., 2002; Millán et al., 2010; Cortés-Campos et al., 2011; Orellana et al., 2012; Salgado et al., 2014; Elizondo-Vega et al., 2016). As GK catalyzes the rate-limiting step of glycolytic metabolism, and high glycolytic flux in the tanycytes allows the release of lactate, we propose that nuclear compartmentalization of GK in high glucose condition may act as a molecular switch to arrest cellular signaling generated by this condition. The nuclear space is readily accessible to low-molecular-mass ligands such as glucose and consequently provides the molecular explanation for the compartmental reshuffling of GK during fasting-feeding transitions. However, it is necessary to demonstrate that there is an *in vivo* molecular interaction of both proteins in tanycytes despite the unfavorable glucose-induced GK conformation. On the other hand, in high glucose concentrations, the activation of pentose phosphate pathway could be necessary for the protection of the oxidative stress such as it has been shown in astroglia (Takahashi et al., 2012). In summary, different metabolic pathways and their potential metabolites, together with the expression ratio of Gckr/Gck cell-type specific, might determine GK and GKRP subcellular localization in response to glucose. Additionally, it will be of great interest enlighten the mechanism that regulates expression of GKRP in hepatocytes and tanycytes, the evolutionary process responsible for the fine-tuning in expression on these cell types, and the minimal expression ratio required to trigger translocation. In relation to the later, Bosco et al. (2000) used an inducible overexpression system that leads to leaky expression of GKRP that was not greater than 2% of GK expression in insulinoma. Interestingly, leaky expression of GKRP was not sufficient to trigger nuclear GK translocation at 0.5 mM glucose, similar to tanycytes.

In one way or another, these results open doors for future studies aiming to understand which biological processes are conducted by tanycytes at high glucose concentrations, which requires GK to be translocated to the nucleus. A compressive transcriptomic analysis of tanycytes, at high and low glucose concentrations, would identify the biological pathways modulated in such metabolic conditions, highlighting potential metabolites that might interact with the complex GK:GKRP in this cell type. In this sense, Single Nucleotide Polymorphisms (SNPs) variants of GCKR has been associated by Genome-wide Association Study (GWAS) to hypertriglyceridemia in humans (Saxena et al., 2007; Orho-Melander et al., 2008; Vaxillaire et al., 2008). It is possible that GKRP is responding to other metabolic processes that might be activated in tanycytes by high glucose concentrations that could be responsible for GK compartmentalization dynamics. However, we cannot rule out the existence of accessory proteins that could be differentially regulating the dynamics of compartmentalization in different cell types.

In summary, we can conclude that the cell-type specific expression ratio of *Gckr*/*Gck* is an important factor to determine the subcellular localization of GK in response to glucose. However, the complete molecular mechanism underlying the compartmentalization dynamics in tanycytes are yet unknown.

## Author Contributions

The experiments were performed at the Department of Cell Biology at the University of Concepcion. MS, ET-S, MG-R, and PO conceived the experiments. MG-R, MS, PO, EU, and MV designed the experiments. MS, PO, and MV performed the experiments. MS, PO, EU, MG-R, and MV analyzed the data. MG-R and EU contribute to reagents, materials and analysis tools. ET-S and MG-R wrote the article. ET-S, MG-R, and EU critically revised the manuscript. All authors have approved the final version of the manuscript and agree to be accountable for all aspects of the work in ensuring that questions related to the accuracy or integrity of any part of the work are appropriately investigated and resolved. All persons designated as authors qualify for authorship, and all those who qualify for authorship are listed.

## Conflict of Interest Statement

The authors declare that the research was conducted in the absence of any commercial or financial relationships that could be construed as a potential conflict of interest.

## Footnotes

1 http://rsb.info.nih.gov/ij/

## Supplementary Material

FIGURE S1 Scheme for adenoviruses preparation. To produce adenoviral particles capable to overexpress GKRP, we subcloned tanycytes-derived Gckr cDNA from H6PQE60 vector **(A)** into pdc311.4-RFP shuttling vector **(G)**. For that, we introduced by PCR 5′-BamHI and 3′-KpnI flanking Gckr sequence to posteriorly clone it the passage vector PCR blunt II TOPO^®^ **(B)**. In parallel, we cloned in frame RFP coding sequence in pcd311.3 vector **(C–F)**, through insertion by PCR of restriction sites 5′KpnI and 3′NotI **(C)**, ligation in TOPO vector **(E)**, and subcloning this sequence in pcd311.3 vector **(F)** by KpnI/NotI digestion a T4 ligase-dependent ligation **(F)**. This last construction was used as a control vector **(H)**. Finally, we have induced recombination of shuttling vector (GKRP overexpressing and control vector) with pBHGloxDE1.3Cre vector, which encodes to structural viral genes lacking the early replication genes E1 and E3, present in HEK 293A cell line.

FIGURE S2 Compartmentalization dynamics of transduced insulinoma cells with Ad-control and no transduced. Non-transduced cells **(A–J)** and transduced cells with Ad-control **(K–T)** were cultured for 6 h with 0.5 mM to posterior increase to 50 mM glucose. **(U,V)** Zoom picture focusing on one cell to show GK and GKRP subcellular localization on Ad-GKRP transduced cells (see Figure 2, main text). Arrowheads are indicating the nucleus of insulinoma cells.

FIGURE S3 Translocation dynamics for GK and GKRP in insulinoma cells overexpressing GKRP at decreasing glucose concentrations. Transduced insulinoma cells were cultured for 3 h at 50 mM glucose with a posterior increase to 0.5 mM glucose for 30 min. **(A)** Dynamic subcellular localization of GKRP through fluorescence detection of RFP. **(B)** Dynamic subcellular localization of GK through immunocytochemistry. Scale Bar 20 μm. **(C,D)** Quantification of nuclear/cytoplasmic ratio measured by fluorescence intensity of both subcellular compartments for GKRP **(C)** and GK **(D)**. Inserted box: Saccharose control treatments. Arrowheads are indicating the nucleus of insulinoma cells.

FIGURE S4 Downregulation of GKRP through shRNA. For downregulation, we constructed adenovirus that overexpress a shRNA for GKRP (Ad-shGKRP-EGFP) and a shRNA for β-galactosidase (Ad-shβgal-EGFP). Transduced tanycytes were culture at 0.5 mM for 6 h with a posterior increased to 15 mM glucose for 30 min. **(A–F)** Subcellular localization of GK in cells cultured with 0.5 mM glucose and transduced with Ad-control **(A–C)** or Ad-shGKRP-EGFP **(D–F)**. **(G–L)** Subcellular localization of GK in cells cultured with 15 mM glucose and transduced with Ad-control **(G–I)** or Ad-shGKRP-EGFP **(J–L)**. Nuclear localization of GK is lost at 15 mM glucose when GKRP is downregulated.

## Abbreviations

Ad-control: adenovirus carrying RFP
Ad-GKRP: adenovirus constructed to overexpressed GKRP
F6P: Fructose-6- phosphate
G-6P: glucose-6-phosphate
*Gck*: gene encoding GK
*Gckr*: gene encoding GKRP
GK: glucokinase
GKRP: glucokinase regulatory protein
GLUT2: glucose transporter 2
KO: Knockout
RFP: Red fluorescent protein
