# Supplementary figures and images for: When a Little Bit More Makes the Difference: Expression Levels of GKRP Determines the Subcellular Localization of GK in Tanycytes
Source: Front Neurosci. 2019 Mar 29;13:275. doi: 10.3389/fnins.2019.00275 (PMC6449865; doi:10.3389/fnins.2019.00275)

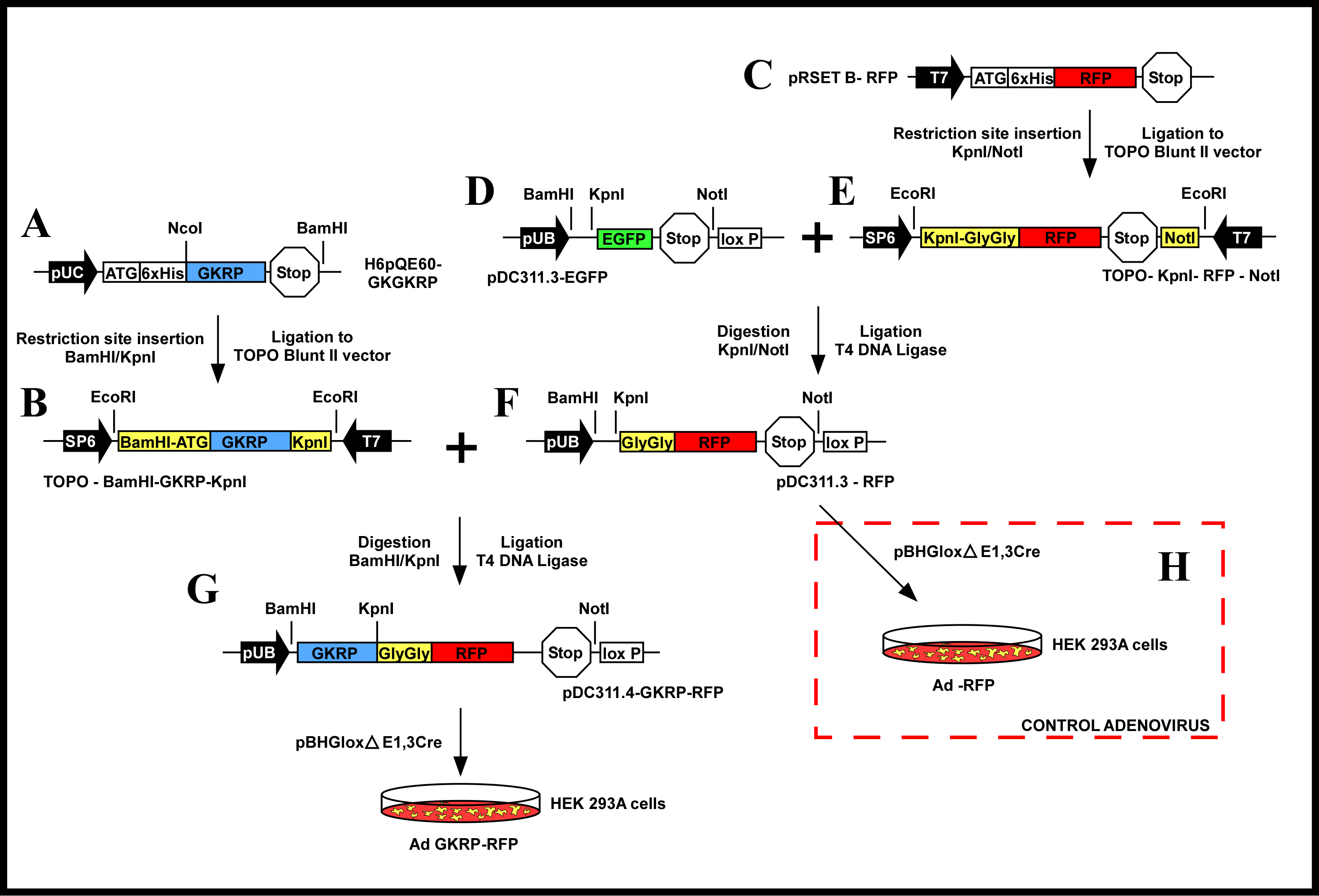

Supplement: FIGURE S1 — Scheme for adenoviruses preparation. To produce adenoviral particles capable to overexpress GKRP, we subcloned tanycytes-derived Gckr cDNA from H6PQE60 vector (A) into pdc311.4-RFP shuttling vector (G). For that, we introduced by PCR 5′-BamHI and 3′-KpnI flanking Gckr sequence to posteriorly clone it the passage vector PCR blunt II TOPO® (B). In parallel, we cloned in frame RFP coding sequence in pcd311.3 vector (C–F), through insertion by PCR of restriction sites 5′KpnI and 3′NotI (C), ligation in TOPO vector (E), and subcloning this sequence in pcd311.3 vector (F) by KpnI/NotI digestion a T4 ligase-dependent ligation (F). This last construction was used as a control vector (H). Finally, we have induced recombination of shuttling vector (GKRP overexpressing and control vector) with pBHGloxDE1.3Cre vector, which encodes to structural viral genes lacking the early replication genes E1 and E3, present in HEK 293A cell line. [file Image_1.TIF]

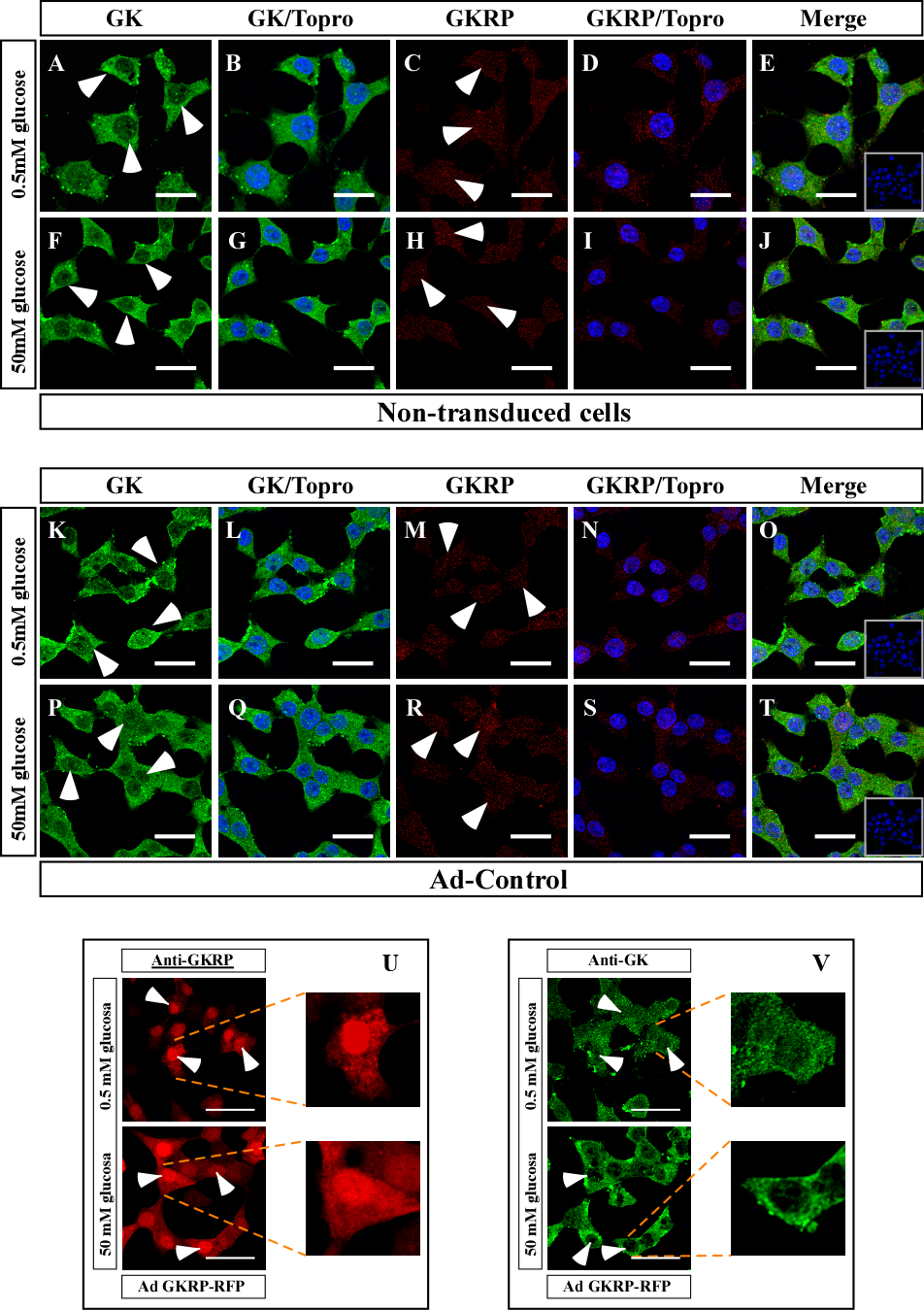

Supplement: FIGURE S2 — Compartmentalization dynamics of transduced insulinoma cells with Ad-control and no transduced. Non-transduced cells (A–J) and transduced cells with Ad-control (K–T) were cultured for 6 h with 0.5 mM to posterior increase to 50 mM glucose. (U,V) Zoom picture focusing on one cell to show GK and GKRP subcellular localization on Ad-GKRP transduced cells (see Figure 2, main text). Arrowheads are indicating the nucleus of insulinoma cells. [file Image_2.TIF]

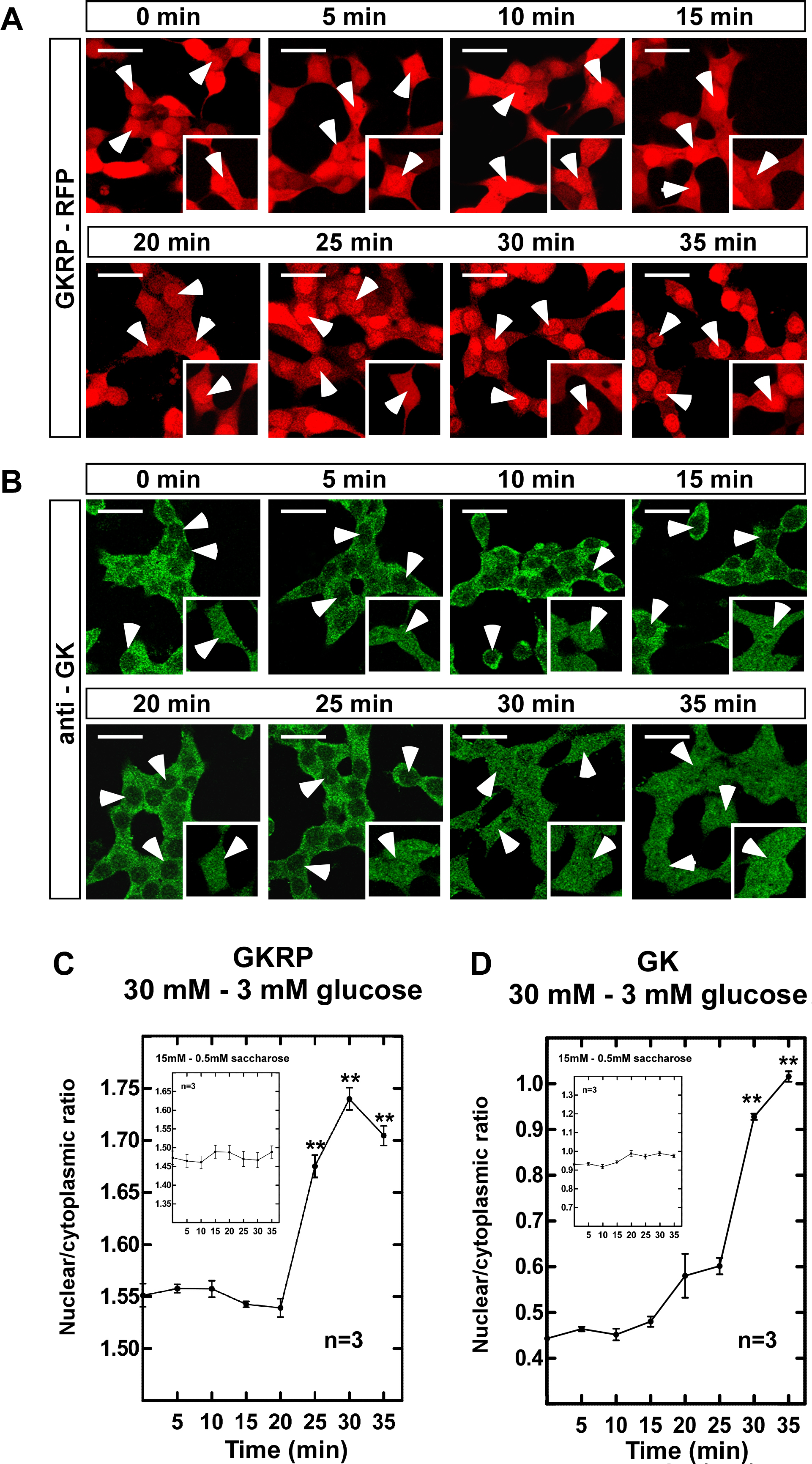

Supplement: FIGURE S3 — Translocation dynamics for GK and GKRP in insulinoma cells overexpressing GKRP at decreasing glucose concentrations. Transduced insulinoma cells were cultured for 3 h at 50 mM glucose with a posterior increase to 0.5 mM glucose for 30 min. (A) Dynamic subcellular localization of GKRP through fluorescence detection of RFP. (B) Dynamic subcellular localization of GK through immunocytochemistry. Scale Bar 20 μm. (C,D) Quantification of nuclear/cytoplasmic ratio measured by fluorescence intensity of both subcellular compartments for GKRP (C) and GK (D). Inserted box: Saccharose control treatments. Arrowheads are indicating the nucleus of insulinoma cells. [file Image_3.TIF]

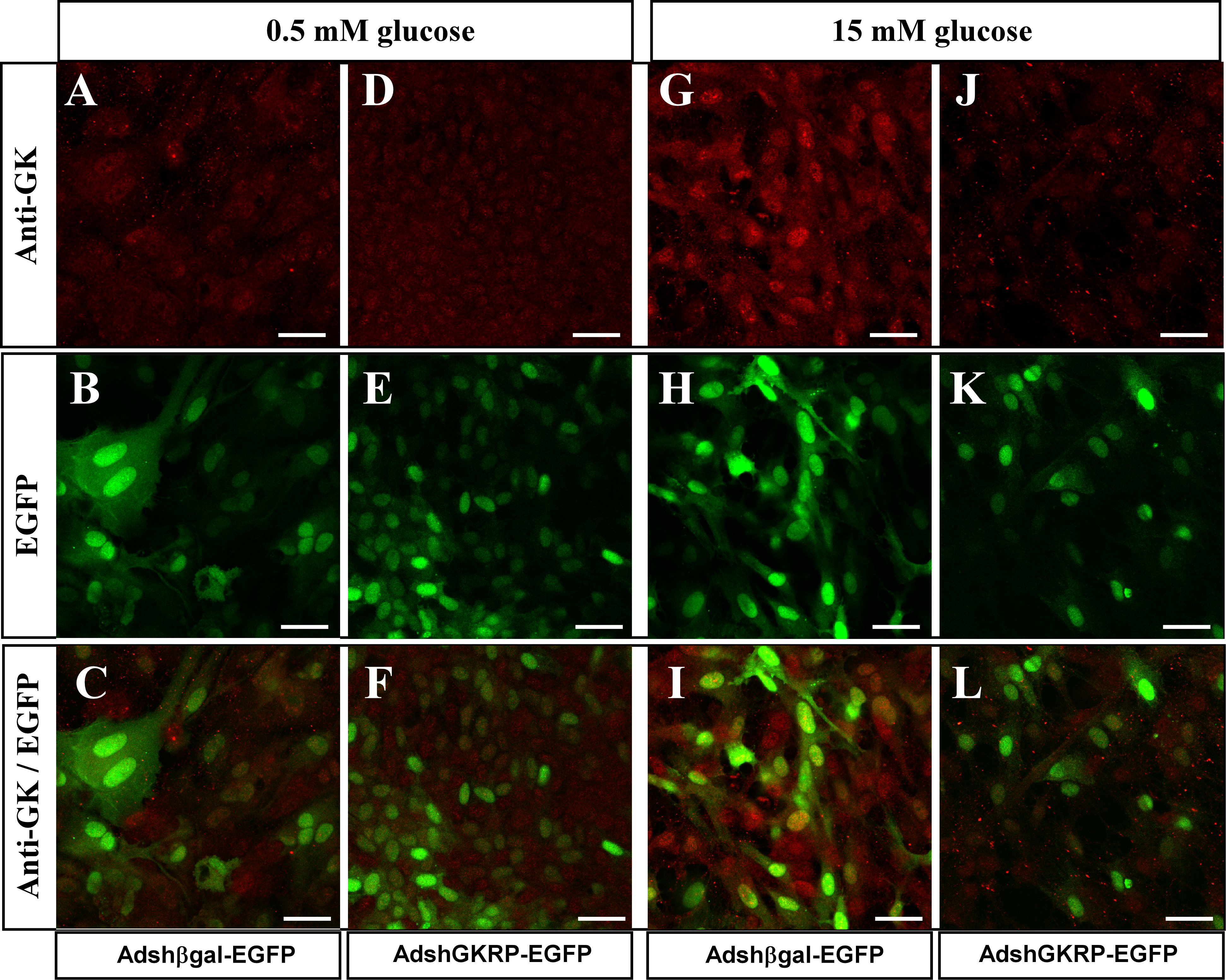

Supplement: FIGURE S4 — Downregulation of GKRP through shRNA. For downregulation, we constructed adenovirus that overexpress a shRNA for GKRP (Ad-shGKRP-EGFP) and a shRNA for β-galactosidase (Ad-shβgal-EGFP). Transduced tanycytes were culture at 0.5 mM for 6 h with a posterior increased to 15 mM glucose for 30 min. (A–F) Subcellular localization of GK in cells cultured with 0.5 mM glucose and transduced with Ad-control (A–C) or Ad-shGKRP-EGFP (D–F). (G–L) Subcellular localization of GK in cells cultured with 15 mM glucose and transduced with Ad-control (G–I) or Ad-shGKRP-EGFP (J–L). Nuclear localization of GK is lost at 15 mM glucose when GKRP is downregulated. [file Image_4.TIF]
